# GluN2B-containing NMDA receptors and AMPA receptors in medial prefrontal cortex are necessary for odor span in rats

**DOI:** 10.3389/fnbeh.2013.00183

**Published:** 2013-12-02

**Authors:** Don A. Davies, Quentin Greba, John G. Howland

**Affiliations:** Department of Physiology, University of SaskatchewanSaskatoon, SK, Canada

**Keywords:** working memory, odor span task, CNQX, Ro 25-6981, CPP, glutamate

## Abstract

Working memory is a type of short-term memory involved in the maintenance and manipulation of information essential for complex cognition. While memory span capacity has been extensively studied in humans as a measure of working memory, it has received considerably less attention in rodents. Our aim was to examine the role of the N-methyl-D-aspartate (NMDA) and α-Amino-3-hydroxy-5-methyl-4-isoxazolepropionic acid (AMPA) glutamate receptors in odor span capacity using systemic injections or infusions of receptor antagonists into the medial prefrontal cortex (mPFC). Long Evans rats were trained on a well-characterized odor span task (OST). Initially, rats were trained to dig for a food reward in sand followed by training on a non-match to sample discrimination using sand scented with household spices. The rats were then required to perform a serial delayed non-match to sample procedure which was their odor span. Systemic injection of the broad spectrum NMDA receptor antagonist 3-(2-Carboxypiperazin-4-yl)propyl-1-phosphonic acid (CPP) (10 mg/kg) or the GluN2B-selective antagonist Ro 25-6981 (10 mg/kg but not 6 mg/kg) significantly reduced odor span capacity. Infusions of the GluN2B- selective antagonist Ro 25-6981 (2.5 μg/hemisphere) into mPFC reduced span capacity, an effect that was nearly significant (*p* = 0.069). Infusions of the AMPA receptor antagonist 6-cyano-7-nitroquinoxaline-2,3-dione (CNQX) (1.25 μg/hemisphere) into mPFC reduced span capacity and latency for the rats to make a choice in the task. These results demonstrate span capacity in rats depends on ionotropic glutamate receptor activation in the mPFC. Further understanding of the circuitry underlying span capacity may aid in the novel therapeutic drug development for persons with working memory impairments as a result of disorders such as schizophrenia and Alzheimer’s disease.

## Introduction

Working memory, a type of short term memory, enables the maintenance and manipulation of information needed for complex cognitive functions (Goldman-Rakic, 1996; Baddeley, 2003; D’Esposito, 2007). Working memory is impaired in numerous brain disorders including schizophrenia (Barch et al., 2009) and Alzheimer’s disease (Huntley and Howard, 2010); thus, the use of appropriate preclinical working memory tasks in rodents to understand the neural mechanisms underlying working memory provides one approach for the development of novel therapeutics. In animals, working memory has been assessed using a variety of different tasks, many of which relate solely to the short-term storage of information over delays, without assessment of the capacity of working memory (Dudchenko, 2004; Dudchenko et al., 2012). In schizophrenia, working memory capacity is decreased (Chey et al., 2002; Gold et al., 2010) and the Cognitive Neuroscience Treatment Research to Improve Cognition in Schizophrenia (CNTRICS) group has identified capacity as a component of working memory requiring more basic research before being included in the translational battery (Barch and Smith, 2008; Dudchenko et al., 2012).

**Figure 1:**
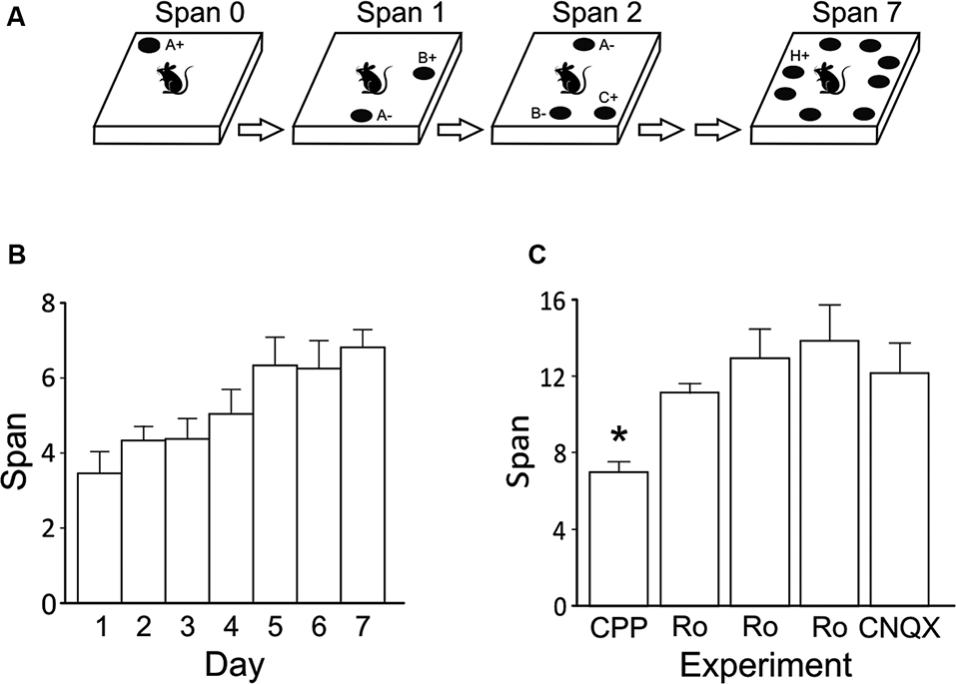
**(A)** Illustration of the OST. See text for details. Odors are indicated with letters. On subsequent trials for a given span, the bowl (black circle) that contains the novel odor is rewarded (+) while all previously encountered stimuli are not (−). Bowls are added one at a time to the platform until an error is made. The span is calculated as the number of bowls on the platform for the last error free trial minus 1. Note that all bowls are moved around the platform before each new trial. **(B)** Mean odor spans during the 7 days of training immediately prior to the first treatment (3-(2-Carboxypiperazin-4-yl)propyl-1-phosphonic acid (CPP); *n* = 8). **(C)** Mean odor spans on the baseline sessions before each treatment in the within subjects design (see Table 1). The GluN2B-selective NMDA receptor antagonist Ro 25-6981 was given three times: first, 6 mg/kg (i.p.); second, 10 mg/kg (i.p.); and third, 2.5 μg/hemisphere. * Refers significantly lower span relative to all other conditions (*p* < 0.05). Ro, Ro 25-6981.

Working memory capacity has been studied in rodents using span tasks with odors or spatial locations as stimuli. One of these tasks is the odor span task (OST) first developed by Dudchenko et al. (2000) (Figure 1A). The OST is an incremental non-match-to-sample task where rats or mice receive a food reward by choosing to dig in a bowl of sand with the novel scent (Dudchenko et al., 2000; Young et al., 2007; Rushforth et al., 2010, 2011; Davies et al., 2013) or by moving scented lids (MacQueen et al., 2011; April et al., 2013; Galizio et al., 2013). Once the subject chooses the novel bowl, additional bowls are added with the previous bowl(s) rearranged on the platform until the subject chooses a previously rewarded bowl. The number of bowls correctly discriminated minus 1 is the span of the subject. Mean spans of approximately 7–9 odors have been reported when rats are stopped after their first error (Dudchenko et al., 2000; but see April et al., 2013; Davies et al., 2013). Span capacity declines following reversible inactivation of the medial prefrontal cortex (mPFC; Davies et al., 2013), but not permanent lesions of dorsal hippocampus (Dudchenko et al., 2000), in rats. Span capacity is also reduced following exposure to acute stress (Davies et al., 2013). Further research has demonstrated that odor span capacity is increased by systemically administered nicotinic receptor agonists (Rushforth et al., 2010) while it is transiently reduced following 192 IgG-saporin-induced cholinergic lesions of the basal forebrain (Turchi and Sarter, 2000). Odor span is also impaired by non-competitive N-methyl-D-aspartate (NMDA) receptor antagonists (MacQueen et al., 2011; Rushforth et al., 2011; Galizio et al., 2013) and the *γ*-aminobutyric acid (GABA) A receptor modulator chlordiazepoxide (Galizio et al., 2013).

**Table 1:** **Schedule of treatments during the experiment**.

**Week**	**Stage**
1	Habituation and dig training
2–3	Non-matching-to-sample
3–4	Odor span training
4–5	CPP (10 mg/kg; i.p.)
6	Ro 25-6981 (6 mg/kg; i.p.)
7	Washout
8	Ro 25-6981 (10 mg/kg; i.p.)
9–10	mPFC cannulae implantation and recovery
11	Ro 25-6981 (2.5 μg/hemisphere; mPFC)
12	CNQX (1.25 μg/hemisphere; mPFC)

*Note that all drug treatments were paired with a vehicle treatment on the same week*.

Limited information exists regarding the effects of brain site-specific modulation of neurotransmitters and their receptors on working memory capacity as assessed by the OST. In one study, working memory capacity was increased in transgenic mice over-expressing the NMDA receptor subunit GluN2B in forebrain areas including the cortex (Cui et al., 2011). A study examining the maintenance, but not capacity, of working memory demonstrates a role of ionotropic glutamate receptors in the dorsolateral prefrontal cortex for working memory in monkeys (Wang et al., 2013). Using the delayed occulomotor response task, Wang et al. (2013) showed that NMDA receptors containing GluN2B subunits in the dorsolateral prefrontal cortex are essential for the maintenance of working memory by regulating the activity of delay period neural activity during the task. Mixed effects were found for the AMPA receptor antagonist 6-cyano-7-nitroquinoxaline-2,3-dione (CNQX) in the same paradigm in monkeys (Wang et al., 2013) and previous studies examining working memory in rodents (Li et al., 1997; Romanides et al., 1999).

Taken together, these findings suggest that ionotropic glutamate receptors in the mPFC may be critical for span capacity. To test this possibility, we used the OST in rats and first performed systemic injections of the broad NMDA receptor antagonist CPP (Lehmann et al., 1987) and the GluN2B-selective antagonist Ro 25-6981 (Fischer et al., 1997). Subsequently, we used direct intracranial infusions of Ro 25-6918 and the AMPA receptor antagonist CNQX (Honore et al., 1988) targeted to the mPFC to specify a role for receptors in that area in odor span capacity.

## Methods

### Animals

Eight adult male Long-Evans rats (270–310 g; Charles Rivers, Quebec, Canada) were tested in the experiments using a within subjects design (Table 1). For 6 days after arrival to the facility, the rats were paired housed in clear plastic cages in a colony room on a 12 h light/dark cycle (lights on at 07:00) with *ad libitum* access to food (Purina Rat Chow) and water. Otherwise, the rats were individually caged with *ad libitum* access to water and were food restricted to maintain 85% of their free feeding weight (except for several days before and after surgery when food was also available *ad libitum*). All experiments were conducted in accordance with the standards of the Canadian Council on Animal Care and were approved by the University of Saskatchewan Animal Research Ethics Board.

### Apparatus and materials

Both training and testing occurred on a 91.5 cm^2^ black corrugated plastic platform with 2.5 cm tall border. The platform was fastened to a metal frame with casters attached and stood 95 cm above the floor. It was surrounded by a beige curtain to block visual cues in the testing room. Velcro was used to fasten the sand-filled bowls to the platform and stop the rats from spilling the sand. Pieces of Velcro were equally spaced along the edge of the platform (one piece in each corner and five additional pieces on each side) and the bowls for a given trial were placed randomly on the pieces of Velcro.

### Odors

Odors were mixed in Premium Play Sand (Quikrete Cement and Concrete Products, Atlanta, GA) and then placed in white porcelain bowls (4.5 cm high, 9 cm in diameter) on the platform as needed for each trial. Sand (100 g) was scented by mixing 0.5 g of a single dried spice. Twenty-four different scents were used in the experiments: allspice, anise seed, basil, caraway, celery seed, cinnamon, cloves (0.1 g), cocoa, coffee, cumin, dill, fennel seed, garlic, ginger, lemon and herb, marjoram, mustard powder, nutmeg, onion powder, orange, oregano, paprika, sage, and thyme. Spices were purchased from a local grocery store. The odors used each day were selected using a random list generator.^1^ All subjects were regularly exposed to all odors.

### Training on the odor span task

Previously published protocols were followed closely (Dudchenko et al., 2000; Davies et al., 2013). Behavioral training was conducted during the light phase with the experimenter blind to the treatments administered. In all phases of the OST, rats were tested 5–7 days per week with treatment days always occurring 24 h after a training day except for the CNQX infusions in which both treatments (vehicle or CNQX) occurred 24 h apart, which was necessary due to constraints involving the availability of personnel. Order effects were not observed in this experiment. Rats were handled once before dig training for 3 min. *Dig Training/Shaping*. First, rats were trained to dig for a cereal reward (Kellogg’s Froot Loops) in a bowl filled with 100 g of unscented sand. Rats were placed opposite to a bowl on the platform for three separate phases. In the first phase, the food reward was positioned on top of the sand, in the second phase the food reward was incompletely buried, and in the third phase, the food reward was fully buried in the sand. Rats were trained until they would consistently dig for the food reward regardless of bowl position on the platform. This phase of training took 6–9 days to complete. *Odor non-matching-to-sample*. Once the rats reliably dug in unscented bowls, they moved onto the non-matching-to-sample task. In the sample phase of a trial, the rat was presented with a bowl of scented sand randomly positioned on the platform. After the rat dug and consumed the food reward, it was removed from the platform and placed behind a curtain to obscure its vision of the platform. The experimenter then positioned the bowl on the opposite end of the platform and added a second bowl with a different odor to the platform. In the choice phase of the trial, the rat was positioned on the platform opposite to both bowls and then allowed access to both bowls. A food reward was only in the bowl with the novel odor for that trial. A choice was scored if the rat dug or placed its paws or nose on the sand and an error was scored if the rats chose the previously rewarded odor. The rats were given six non-matching-to-sample trials each day until they chose the novel odor on five of the six trials for 3 days.

### Odor span task

After performing the non-matching-to-sample task reliably, rats were introduced to the OST. Trials were run as described for the non-matching-to-sample task except that bowls with novel odors (for that trial) were added and previous bowls remained until the rat made an error (i.e., dug in any of the bowls except the novel one) which resulted in the trial ending. Previously presented bowls were randomly repositioned before each novel bowl was added to the platform. Therefore, rats could not use spatial cues to guide their choice of bowl. The span for a given trial was scored as the number of odor bowls correctly chosen minus 1 bowl. During training, four rats were transported to the testing room together. Each rat performed 2 or 3 “spans” per day (rats with high spans performed 2 spans while rats with low spans performed 3 spans) with a break between spans occurring while the other rats were tested. Averaging the spans reduced the variance in our sample as reported with other memory tests (Winters and Reid, 2010). The mean of all spans for a given day is reported in the figures. Once performance reached a span of 7 for two training days (8–16 days of training), the pharmacological treatments began. On treatment days, rats were tested for approximately 30 min (1–3 spans) without a break between spans.

### Probe sessions

To determine if the rats were using the odor to solve the task, two types of probe sessions were conducted. The first probe session was implemented to test if the scent of the food reward guided behavior. In this session (3 bowls on the platform: span of 2), the rats were presented with bowls as described in the OST with the exception that the food reward was absent from all bowls. When the rat made a correct choice, the experimenter dropped a food reward on top of the sand in the correct bowl. The second probe was implemented to test if the rats were marking the bowls of sand when they examined them. During a phase in this probe session (2 bowls on the platform: span 1), all of the bowls and sand were replaced with new bowls and new sand that contained the same odors. If rats were marking bowls and sand, their performance would be reduced on these trials. The rats’ performance was 100% accurate during both of these probe sessions (data not shown).

### Systemic drug administration

Rats were injected 30 min prior to starting the OST. For the CPP experiment, rats were injected (i.p.) with either vehicle (saline) or CPP (10 mg/kg; i.p.). This dose was chosen on the basis of previous studies (Whitlock et al., 2006). For the Ro 25-6981 experiment, rats were injected (i.p.) with either vehicle (20% DMSO; 80% H_2_O) or Ro 25-6981 (6 mg/kg or 10 mg/kg; i.p.; Wong et al., 2007; Howland and Cazakoff, 2010; Li et al., 2010).

### Surgery and mPFC infusions

Surgeries for the mPFC and OST experiment were conducted after the systemic Ro 25-6981 (10 mg/kg) injections experiment. Subjects were anesthetized with isoflurane and prepared for surgery using previously reported procedures (Cazakoff and Howland, 2011; Davies et al., 2013). Guide cannulae (23 Ga) were bilaterally inserted above mPFC (AP + 2.60 mm; ML ± 0.70 mm; DV −3.60 mm; flat skull). Obdurators (0.033 cm diameter stainless steel wire) were placed into the cannulae to avoid obstruction. Following surgery, rats were allowed to recover for 8 days before training resumed. One rat died during surgery. Thus, the *n* for the infusion experiments is seven.

Rats were habituated to the infusion procedure on three different days during the week before infusions were administered (Cazakoff and Howland, 2011; Davies et al., 2013). Infusions were achieved by inserting custom made needles (30 Ga stainless steel tubing) linked via PE-50 tubing to an infusion pump (PHD 2000, Harvard Apparatus, Holliston, MA) 1 mm past the end of the cannulae. Needles were inserted into both cannula then delivery of Ro 25-6981, CNQX or the vehicle into the mPFC was initiated (0.5 μl in 1 min). The infusion needles were left in place for an additional minute after the infusion to permit diffusion of the drug. Rats were tested on the OST 15 min following brain infusions. Ro 25-6981 (2.5 μg/0.5 μl; Zhang et al., 2008) and vehicle (12% DMSO; 88% PBS) were given in a counterbalanced order followed by CNQX (1.25 μg/0.5 μl; Ho et al., 2011) and vehicle (PBS) which were also counterbalanced.

Following testing in all conditions, the rats were sacrificed with isoflurane and perfused with saline. Brains were removed and post-fixed in a 10% formalin-10% sucrose solution. Brains were sectioned on a sliding microtome and infusion sites were determined using standard protocols with reference to a rat brain atlas (Paxinos and Watson, 1997).

### Data analysis

Odor spans and latencies to choose the first bowl were manually recorded during testing and entered into Microsoft Excel (2010) and Statistical Package for the Social Sciences (SPSS; version 19.0) for analysis. All descriptive values are reported as means ± standard error of the mean. Comparisons were done using paired *t*-tests. Analysis of the spans for baseline sessions before the various treatments were compared using a repeated measures ANOVA followed by Neuman Keuls post-hoc tests (Figure 1C). Statistical tests were considered significant if *p* values were < 0.05.

## Results

### Training

After dig training, we trained rats in the non-matching-to-sample task until they selected the novel odor in 5/6 trials for three sessions (mean = 7.13 days; range = 6–9 days). Rats were then trained on the OST (Figure 1A) for a mean of 12.38 sessions (range, 8–16 days). During the 7 days immediately prior to the first treatment, the rats reached a span of approximately 7 odors (Figure 1B), as previously reported when sand-filled bowls are used (Rushforth et al., 2010, 2011; Davies et al., 2013).

Figure 1C displays the spans of the rats’ baseline sessions. Over the 8 weeks of repeated testing (Table 1), we noticed a significant increase in span on the baseline sessions (*F*(4, 28) = 7.06, *p* < 0.001). Average baseline spans increased from a mean of 7.00 ± 0.5 the day before CPP treatment to 12.14 ± 1.6 before CNQX treatment. It should be noted that surgery was performed on the rats between the second systemic administration of Ro 25-6918 and first intracranial injection of Ro 25-6981 into the mPFC. Surgery had no measureable effect on the average span observed following the retraining period (two sessions).

### Systemic injection of either CPP or Ro 25-6981 impairs odor span

**Figure 2:**
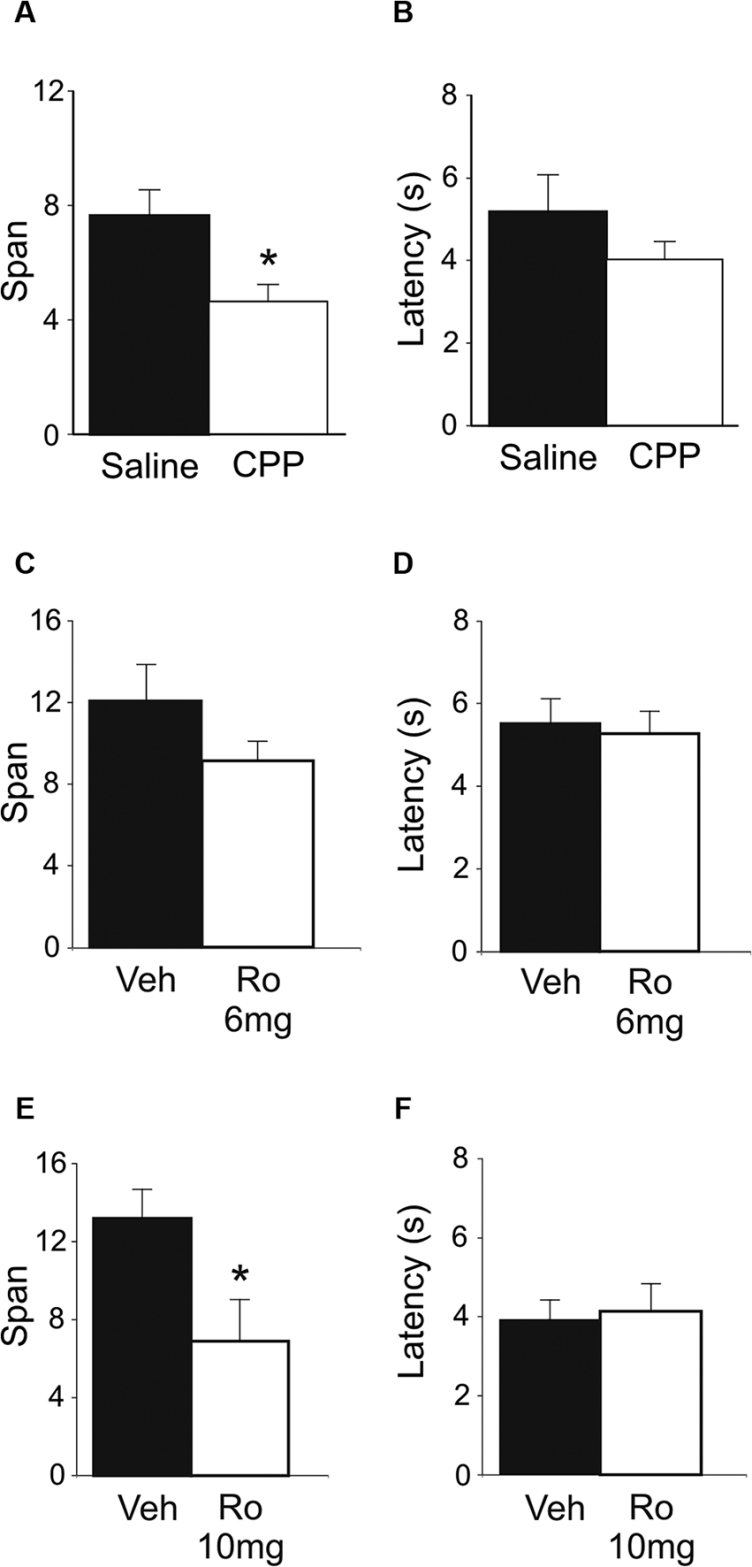
**Effects of systemic NMDA receptor antagonism on performance of the OST (*n* = 8). (A)** Mean spans of the rats following saline or CPP treatment (10 mg/kg, i.p.). **(B)** The mean latency of the rats to begin digging in a bowl (saline or CPP). **(C)** Mean spans for the rats following vehicle (Veh) or Ro 25-6981 (Ro) treatment (6 mg/kg, i.p.) **(D)** The mean latency of the rats to begin digging in a bowl during the tests conducted in C. **(E)** Mean spans for the rats following each treatment with either Veh or Ro 25-6981 (10 mg/kg, i.p.). **(F)** The mean latency of the rats to begin digging in a bowl (Veh or Ro, 10 mg/kg). * Refers to a significant difference between treatments (*p* < 0.05).

Systemic injections of CPP (10 mg/kg; i.p.) impaired span capacity (Figure 2A) without affecting latency to dig in the OST (Figure 2B). Following CPP rats had a span of 4.64 ± 0.6 odors which was significantly lower than when the rats were injected with saline (7.65 ± 0.9 odors; *t*(7) = 5.19, *p* < 0.001). Latencies to begin digging in bowls did not differ between treatments (Figure 2B; Saline = 5.17 ± 0.9 s; CPP = 4.03 ± 0.4 s; *t*(7) = 1.12, n.s.).

To test the effect of blocking only NMDA receptors containing GluN2B subunits, two doses of Ro 25-6981 were administered systemically (i.p.). A trend of decreased odor span was found following administration of the 6 mg/kg dose (Figure 2C; Veh = 12.07 ± 1.8 odors; Ro 25-6981 = 9.14 ± 1.0); however, this difference was not significant (*t*(7) = 1.41, n.s.). A higher dose of Ro 25-6981 (10 mg/kg) significantly reduced span in rats (Figure 2E; Veh = 13.19 ± 1.5; Ro 25-6981 = 6.90 ± 2.2; *t*(7) = 3.55, *p* = 0.009). Latency for the rats to begin digging in a bowl did not differ between treatments for either dose (Figure 2D *t*(7) = 0.32, n.s.; Figure 2F *t*(7) = −0.42, n.s.). Thus, systemic blockade of GluN2B-containing NMDA receptors impaired odor span capacity without affecting latency in the OST.

### Ro 25-6981 or CNQX infusions in mPFC impair performance of the OST

**Figure 3:**
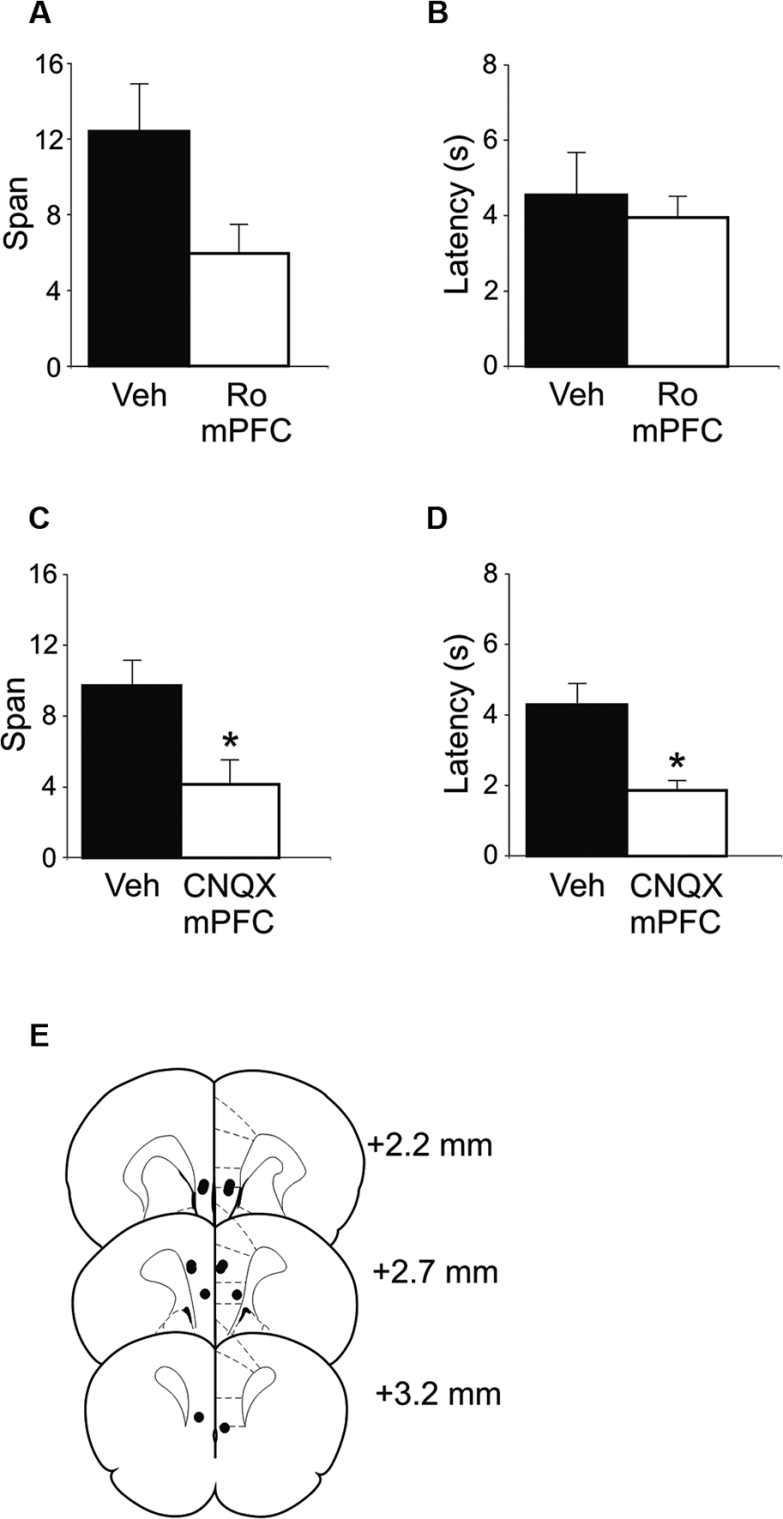
**OST performance following infusions of either Ro 25-6981 or CNQX into mPFC (*n* = 7). (A)** Mean spans of the rats following vehicle (Veh) or Ro 25-6981 infusions into mPFC. **(B)** The mean latency of the rats to begin digging in a bowl for the treatments in A. **(C)** Mean spans for the rats following treatment with either Veh or CNQX into the mPFC. **(D)** The mean latency for the rats to begin digging in a bowl (Veh or CNQX; mPFC infusion). **(E)** Infusion sites in the mPFC. Numbers refer to the anterior-posterior location of the plates relative to bregma. * Refers to a significant difference between treatments (*p* < 0.05).

In an effort to delineate the critical brain regions that underlie disruption in odor span observed following systemic administration of NMDA receptor antagonists, mPFC infusions of Ro 25-6981 were performed. Intra-mPFC Ro 25-6981 infusions impaired span capacity but not latency on the OST (Figures 3A, B). A robust decrease in span was observed in six out of the seven rats. When all seven rats were considered, spans decreased from 12.43 ± 2.5 for vehicle infusion to 5.95 ± 1.6 for Ro 25-6981 infusion (Figure 3A), an approximate 50% decrease in mean span. This difference was close to significant (*t*(6) = 2.21, *p* = 0.069). When the rat that showed the opposite pattern of behavior was removed, a significant difference was observed (Veh = 14.08 ± 2.3; Ro 25-6981 = 4.95 ± 1.4, *t*(5) = 6.32, *p* < 0.001). The latency for rats to begin digging into bowls did not differ between the two treatments (Figure 3B; *t*(6) = −0.40, n.s.).

To test the potential role of mPFC AMPA receptors in span capacity, CNQX was infused into the mPFC (Figures 3C, D). A marked reduction in odor span was observed in rats following CNQX infusions (Veh = 9.71 ± 1.5; CNQX = 4.14 ± 1.4; *t*(6) = 5.57, *p* < 0.001). Latency for rats to begin digging into bowls following CNQX infusions into the mPFC was also significantly reduced by CNQX infusions (Veh = 4.29 ± 0.6 s; CNQX = 1.86 ± 0.3 s; *t*(6) = 3.23, *p* = 0.018).

Figure 3E shows the infusion sites of the rats in the mPFC OST experiments. Infusion sites were located in the prelimbic, infralimbic, and dorsal peduncular areas of the mPFC.

## Discussion

The present study revealed a series of novel findings: (1) span capacity of untreated rats significantly increased from a mean of approximately 7 to a mean of approximately 13 following the first treatment (Figure 1C); (2) systemic injections of the broad spectrum NMDA receptor antagonist CPP significantly reduced span capacity (Figure 2A); (3) systemically administered Ro 25-6981 dose-dependently impaired odor span (Figures 2C, E); (4) GluN2B subunit-containing NMDA receptors in the mPFC may be involved in performance of the OST because Ro 25-6981 infusions into the mPFC marginally impaired span capacity (Figure 3A); (5) blocking AMPA receptors in mPFC with CNQX infusions impaired span capacity and reduced latency to dig in the task compared to vehicle infusions (Figures 3C, D).

### Performance of rats on the odor span task

Rats in the present experiment initially performed similarly to those reported in a previous publication from our group (Davies et al., 2013) and others using the version of the OST that requires the rats to dig in scented sand (Rushforth et al., 2010, 2011). By testing the rats on 1–3 spans per day for a maximum of 30 min, within-session variability was reduced in our study, a characteristic of the OST that has been discussed previously (Dudchenko et al., 2000). We did not observe a consistent pattern of span length over the spans tested on a given day, although it should be emphasized that rats with high spans would not receive a 2nd or 3rd span on a given day given the time constraint (30 min testing/day) we imposed. The results from the pharmacological experiments include the mean of all spans tested for each animal on a given day. If just the first span is considered for the baseline and treatment days, all results are the same as those reported for the mean daily spans (data not shown).

Unexpectedly, the mean baseline spans of the rats in the present study increased to approximately 12 odors with further training (Figure 1C) and as a result performance was not stable before the systemic CPP treatment was given. While it is not clear why a higher span capacity was achieved in the present experiments, probe sessions showed that rats were not using either the odor of the buried food or some unknown feature of the bowls to solve the task. Rats in our previous study (Davies et al., 2013) underwent the same stereotaxic surgery to implant cannulae in the mPFC; thus, effects related to that procedure are likely not involved. The present experiment was conducted in a new facility (with the identical platform and bowls) as our previous report, although the specific reasons why span capacity would be altered in the new facility are unclear. In any case, performance of the rats in the present study is within the normal range for our method of training the OST. When a testing procedure is used that allows rats to continue sampling odors after an error is committed, spans higher than 15 have been reported (Dudchenko et al., 2000; Turchi and Sarter, 2000). Results using a different version of the OST that limits the number of stimuli available on each trial also suggests that maximum span capacity is higher than the previously reported 7–9 odors (April et al., 2013), a result we would expect with that procedure.

This group of rats may have also become better able to recognize the 24 odors used in the task over the extended training they received. On a given day, the odors used for a given span are randomly selected but the same odors are used repeatedly over the weeks of training. Thus, the experience of the rats with the odors may influence their performance. However, this experiment did not assess if the increase of span capacity was due to familiarly with the odors, continuing rule learning, or strengthening of memory. One future test of this hypothesis would be to introduce rats to a series of novel odors after extended training as we conducted in this experiment. If the familiarity of the rats with the “well-trained” odors affected their performance, a reduction in span may be observed when the rats were tested with a series of entirely novel odors.

### Ionotropic glutamate receptors in mPFC are necessary for odor span

The impairment of odor span following treatment with the NMDA receptor antagonist CPP (Figure 2A) is consistent with previously reported effects of the non-competitive NMDA receptor antagonists MK-801 (acute treatment; MacQueen et al., 2011; Galizio et al., 2013) and ketamine (repeated injections) on odor span (Rushforth et al., 2011). Impairments in aspects of working memory other than capacity are well documented following treatment with NMDA receptor antagonists (Li et al., 1997; Doyle et al., 1998; Moghaddam and Adams, 1998; Aura and Riekkinen, 1999; Smith et al., 2011).

The role of NMDA receptor subtypes in cognition has been the subject of intense investigation since it was first shown GluN2B receptors mediate long term depression (LTD) and GluN2A receptors mediate LTP in the hippocampus (Liu et al., 2004). For the first time, we demonstrate that blocking NMDA receptors specifically containing GluN2B subunits systemically impairs odor span capacity (Figure 2E) while infusions of Ro 25-6981 in mPFC marginally impair odor span capacity (Figure 3A). We used the compound Ro 25-6981 which is 5,000 times more selective for GluN2B subunits compared to GluN2A subunits (Fischer et al., 1997). Thus, while we cannot exclude the possibility that some GluN2A-containing NMDA receptors were affected, it is highly likely that the effects we observed on odor span were due to effects of Ro 25-6981 on GluN2B-containing receptors. Previously, we found a systemic dose of 6 mg/kg of Ro 25-6981 was sufficient to prevent the stress-induced disruptions of spatial (Wong et al., 2007; Howland and Cazakoff, 2010) and object memory retrieval (Howland and Cazakoff, 2010). Differences between the presumed site of drug action (hippocampus vs. mPFC) or the specific cognitive operations examined (spatial and recognition memory vs. working memory) may have contributed to the results observed at the dose of 6 mg/kg. Higher doses, including 10 mg/kg, also cause behavioral changes in the forced swim test (Li et al., 2010). Our results compliment a study from Cui and colleagues showing increased span capacity in mice with overexpression of GluN2B-containing NMDA receptors in the forebrain (Cui et al., 2011). Others have demonstrated a role for mPFC GluN2B receptors in trace fear conditioning, which is similar to working memory as it involves a temporal gap between the conditioned and unconditioned stimuli (Gilmartin and Helmstetter, 2010; Gilmartin et al., 2013). However, at least two reports have failed to observe deficits in working memory as assessed using operant delayed-match-to-position paradigms following systemic administration of GluN2B antagonists (Doyle et al., 1998; Smith et al., 2011).

The role of GluN2B-containing NMDA receptors in mPFC neural activity is under investigation. In rodent hippocampus, GluN2B-containing NMDA receptors may be more frequently localized to extrasynaptic areas and become activated when extracellular glutamate levels are elevated such as during acute stress (Yang et al., 2005; Wong et al., 2007; Howland and Wang, 2008). In rodent mPFC, NMDA receptors containing GluN2B subunits are found on pyramidal cells and interneurons and thus may be important for cognitive functions, including working memory. Broad spectrum NMDA receptor antagonists alter the firing properties of mPFC pyramidal cells in rodents by reducing burst firing (Jackson et al., 2004) while increasing basal firing rate (Jackson et al., 2004; Homayoun and Moghaddam, 2007). Interestingly, GluN2A receptors have been shown to critically modulate the increased gamma oscillations observed in cortex following NMDA receptor blockade (Kocsis, 2012). In monkeys, GluN2B-containing receptors are located in the synapses of pyramidal neurons (Wang et al., 2013). Using electrophysiological recordings in freely behaving monkeys, direct application of Ro 25-6981 to the dorsolateral PFC was shown to impair performance of the delayed occulomotor response task and reduce firing of delay neurons in dorsolateral PFC (Wang et al., 2013). Similar results were observed following the systemic administration of ketamine (Wang et al., 2013). Whether similar effects of GluN2B antagonists on neural activity are observed in rodents is difficult to predict as response neurons are more commonly found in the rodent mPFC (Caetano et al., 2012; but see Devilbiss et al., 2012).

In the final experiment, we show that blocking mPFC AMPA receptors impairs span capacity (Figure 3C) while also reducing the latency for rats to make a choice. In previous studies, the effects of manipulating AMPA receptor activity in the mPFC on working memory have been inconsistent. Medial PFC infusions of CNQX impair working memory assessed by delayed alternation (Romanides et al., 1999) while systemic administration of the AMPA receptor antagonist with YM90K failed to alter performance of a radial arm maze task with or without a delay (Li et al., 1997). Application of CNQX to the dorsolateral prefrontal cortex in monkeys produced mixed results on neural activity in the delayed occulomotor response task (Wang et al., 2013). CNQX significantly reduced the firing rate of cue cells; however, it had varied results on response and delay cells. In addition, when rats were treated with CNQX in the OST, they showed reduced latency to dig, which may result from a psychomotor effect from CNQX; thus, caution is warranted when interpreting the impaired span capacity data. Whether this finding reflects increased impulsivity is unclear; however, AMPA receptor blockade in the infralimbic sub-region of the mPFC does not cause an impulsive phenotype in the five choice serial reaction time task (Murphy et al., 2012).

## Conclusion

Previous research using various tasks including the OST implicates the mPFC as an essential substrate for working memory (Kolb, 1991; Seamans et al., 1995; Floresco et al., 1997; Aujla and Beninger, 2001; Davies et al., 2013). The present results suggest a role for GluN2B-containing NMDA receptors and AMPA receptors in the mPFC for span capacity. Caution is warranted regarding the involvement of AMPA receptors in the mPFC during the OST given that both span capacity and latency decreased with CNQX infusions. The CNTRICS group has specified working memory capacity as a construct that needs more basic research before being incorporated into translational sequence for drug development (Barch and Smith, 2008; Dudchenko et al., 2012). Thus, these results may aid in novel therapeutic development for persons with schizophrenia.

## Author contributions

Don A. Davies designed the experiments, conducted research, analyzed data, and co-wrote the manuscript. Quentin Greba conducted research. John G. Howland designed the experiments, co-wrote the manuscript, and supervised the project.

## Conflict of interest statement

The authors declare that the research was conducted in the absence of any commercial or financial relationships that could be construed as a potential conflict of interest.

## Footnotes

^1^http://www.random.org/lists/
